# Sustainable cooling solutions in Dubai: the impact of incident radiation and panel angles on solar AC performance

**DOI:** 10.1038/s41598-026-36069-1

**Published:** 2026-01-22

**Authors:** Sampath Suranjan Salins, Shiva Kumar, Krishna Prasad

**Affiliations:** 1 Manipal Academy of Higher Education, Dubai Campus, Dubai, 345050 UAE; 2 Manipal Institute of Technology, Manipal Academy of Higher Education, Manipal, 576104 India

**Keywords:** Thermal comfort, Sloar panel tilt angle, Cooling performance, Predicted mean vote, Predicted percentage of dissatisfied, Solar COP, Climate sciences, Energy science and technology, Engineering, Environmental sciences

## Abstract

The rising demand for air conditioning units driven by climate change underscores the importance of using sustainable energy sources, which help reduce greenhouse gas emissions and mitigate environmental impact. The present work focuses on the design and construction of a solar air conditioning system by integrating photovoltaic panels or solar thermal collectors to power the cooling cycle, providing a sustainable and eco-friendly alternative to conventional systems. A solar air conditioning unit entails the design, integration, and performance evaluation of a system that harnesses solar energy to provide space cooling. This study focuses on the design and construction of a solar air conditioning unit for cooling a defined space, with performance evaluated under varying solar radiation levels and panel tilt angles. Thermal comfort parameters and power factors were analyzed to determine the system’s overall efficiency. Experiments were carried out under Dubai’s climatic conditions by varying the incident solar radiation from 700 to 1400 W/m^2^ and adjusting the panel inclination angle between 15° and 25° which determined the performance parameters and the thermal comfort. The unit achieved maximum values for moisture removal rate, thermal efficiency, and solar coefficient of performance at 0.74 g/s, 95%, and 1.03, respectively. Power ratios were observed to decrease with increasing incident radiation. A solar panel tilt angle of 25° yielded the highest moisture removal rate 0.78 g/s, solar coefficient of performance 1.1, and solar direct consumption ratio 0.61. Thermal comfort parameters, including the Predicted Mean Vote (PMV) and Predicted Percentage of Dissatisfied (PPD), were calculated to be − 0.21 and 12.7%, respectively, both falling within acceptable comfort ranges.

## Introduction

The increasing global emphasis on renewable energy highlights the critical role of sustainable power generation systems. Among various renewable sources, solar energy has gained considerable attention due to its abundance and clean nature. In regions such as Dubai, where extreme temperatures and intense solar radiation prevail throughout the year, cooling demands dominate total electricity consumption. Conventional air conditioning (AC) systems contribute significantly to grid stress, fossil fuel dependency, and greenhouse gas emissions. Therefore, the development of solar-assisted air conditioning systems offers a promising pathway toward energy efficiency and environmental sustainability.

The present study focuses on evaluating the feasibility and performance of solar-powered air conditioning systems under Dubai’s climatic conditions, with particular attention to energy consumption, indoor thermal comfort, and sustainability objectives^1^. Although photovoltaic (PV) and solar thermal technologies have demonstrated economic benefits, their real-world efficiencies remain limited. For instance, the electrical efficiency of PV panels seldom exceeds 29% due to environmental, material, and operational constraints^2,3^. In Dubai, solar radiation intensity can reach peaks of approximately 1400 W/m^2^ during summer, though it fluctuates significantly throughout the day^4–6^. To ensure optimal energy capture, PV modules must be positioned to minimize shading and receive maximum direct solar irradiance. While solar tracking systems can achieve this, they are often costly and maintenance-intensive. Hence, a fixed installation with an optimized tilt angle provides a practical and economical alternative^5,7^.

### Solar panel tilt angle and radiation

A substantial body of research has examined the effect of solar incident radiation and tilt angle on PV system performance and, by extension, solar cooling systems. Gholami and Nils Røstvik^8^ analyzed PV performance in urban settings and observed that south-facing panels retained higher efficiencies (15%) than north-facing ones (12%) due to differential exposure to solar spectra. Alqurashi et al.^9^ introduced a poly-tilted segmented panel (PTSP) concept to regulate the Solar Radiation Reception Rate (SRRR) in Makkah, finding SRRR values of 59.04–72.32% for tilt angles between 0° and 45°. Khan et al.^10^ optimized tilt angles for monocrystalline silicon PV arrays across Saudi Arabia, identifying 35° as the ideal angle to achieve 86,756.85 Wh/m²/year of incident energy. Similarly, Masili and Ventura^11^ found a 23° tilt to be optimal for Brazilian conditions, noting a decline in performance with higher inclination. Alqaed et al.^12^ determined that the optimal tilt angle for Najran City was 20.9° in summer, increasing slightly during winter in accordance with solar geometry. Ferreira et al.^13^ compared different tracking configurations—including polar, biaxial, and fixed—and concluded that polar and biaxial tracking systems yielded the highest energy capture. Wu et al.^14^ proposed an Incident Solar Radiation Prediction Index (ISRPI), demonstrating that urban geometry strongly affects building-envelope irradiation. Kafka and Miller^15^ developed the Dual Angle Solar Harvest (DASH) concept, combining two different tilt angles to enhance solar capture per unit area, outperforming single-tilt systems. Most studies report optimal tilt angles based on geographical or seasonal averages but lack experimental validation under real operating conditions. Few studies connect tilt angle optimization directly to cooling system performance and indoor comfort.

### Cooling techniques for PV panels

In addition to geometric optimization, several studies have investigated PV cooling methods to alleviate thermal losses. Sivakumar et al.^16^ evaluated water-immersion cooling for PV modules, reporting that immersing panels 20 mm deep in water improved efficiency to 15.54%, outperforming both shallower and deeper configurations. Ganesan et al.^17^ applied neem oil as a phase-change material (PCM) at the rear surface of PV panels, achieving temperature reductions of 2.29% (monocrystalline) and 4.34% (polycrystalline), which enhanced panel efficiencies by 15–18%. Garni^18^ observed that sand and dust storms can degrade PV output by up to 50%, emphasizing the importance of periodic cleaning and water cooling. Hassan et al.^19^ used a copper sulphate layer to enhance surface heat dissipation, reducing temperature by 10 °C and improving overall power output, particularly between 12:00 and 13:00 h.

### Thermal comfort

Thermal comfort has also been a focal research area within solar air-conditioning systems. Abdelgaied et al.^20^ integrated a solid desiccant dehumidifier and a thermal recovery unit, achieving a 28% reduction in energy use, a coefficient of performance (COP) of 1.1, and a solar fraction of 0.65. Reda et al.^21^ developed a solar-driven chiller system using a 58 m^2^ collector array, successfully reducing water temperature from 35 °C to 10 °C, achieving a cooling power of 8 kW and a COP of 0.6. Akyüz et al.^22^ tested a solar AC setup in residential buildings, reporting 8–28% energy savings while maintaining thermal comfort.

### System performance and hybrid configurations

From a performance optimization standpoint, several researchers have examined hybrid and on-grid/off-grid system configurations. Aguilar et al.^23^ analyzed a solar on-grid air-conditioning system in Spain’s Mediterranean climate and found an energy efficiency ratio (EER) of 5.6, noting that severe climatic conditions raised operational costs. Kaneesamkandi and Sayeed^24^ proposed a hybrid solar cooling system combining absorption and vapor-compression cycles, which enhanced COP by 83%, thermal efficiency to 84%, and cooling capacity by 68%, while reducing carbon emissions by 166.4%. Ayadi et al.^25^ tested a 2.67 kW PV-powered AC system in Jordan under both grid-connected and stand-alone modes. The system achieved a 70% operational uptime, with payback periods of 6.9 years (on-grid) and 6.4 years (off-grid). Zayed et al.^26^ examined a solar PV-driven thermoelectric air-conditioning (SPVTEAC) system in Egypt, supported by AI-based predictive modeling using RVFLN-WWO, maintaining an indoor temperature of 27.5 °C with a COP of 1.14. Kumar et al.^27^ evaluated a solar-powered VISI cooler with and without PCM, finding that PCM integration reduced power consumption from 48 W to 40 W, optimized compressor performance, and improved system stability. Ali et al.^28^ investigated a dual-cooling technique for PV modules affected by bird droppings, showing that the method reduced panel surface temperatures by nearly 48%, raised voltage and current outputs by 7–9%, and improved efficiency from 15% to 20%. While hybrid configurations improve efficiency, most studies are location-specific and do not provide a general framework for combining PV performance, cooling efficiency, and tilt optimization. Few studies examine all factors under high-radiation climates like Dubai.

After conducting an extensive literature survey, the following research gaps are identified.

### Research gaps

1. Although extensive research has been conducted on solar air conditioning units, studies specifically addressing the impact of incident solar radiation on their performance parameters remain limited.
2. The impact of solar panel tilt on the performance of solar air conditioning units has not been extensively explored.
3. Energy efficiency & thermal comfort conditions resulting from variations in solar parameters have not been addressed in the existing literature on solar air conditioning units.

### Objectives

1. Design and construction of the solar AC unit to cool the defined space at different times of the day.
2. Evaluation of the solar AC parameters varying with the incident solar radiation and with the solar panel tilted angle.
3. Determination of the thermal comfort parameters and power factors for the varied operating conditions.

### Novelty of the current study

1. Location-specific solar AC design: Unlike previous studies conducted in other regions, the present work focuses on designing and evaluating a solar air conditioning system specifically for Dubai’s high-radiation, high-temperature climate. This ensures that the findings are directly applicable to extreme hot-arid environments, where conventional guidelines may not be accurate.
2. Optimization of tilt angle and radiation capture: While the effect of tilt angle and incident radiation has been studied previously, most research relies on generalized approximations or simulations. In contrast, this study provides experimental validation under real operating conditions in Dubai, identifying the optimal tilt angle that maximizes PV output and enhances cooling performance.
3. Integration of thermal comfort indicators: Previous studies primarily focus on energy efficiency or PV performance. This study introduces thermal comfort metrics (PMV and PPD) as part of the evaluation, linking PV performance and system operation directly to indoor human comfort, which is a novel addition to the existing body of research.

### Methodology

**Fig. 1:**
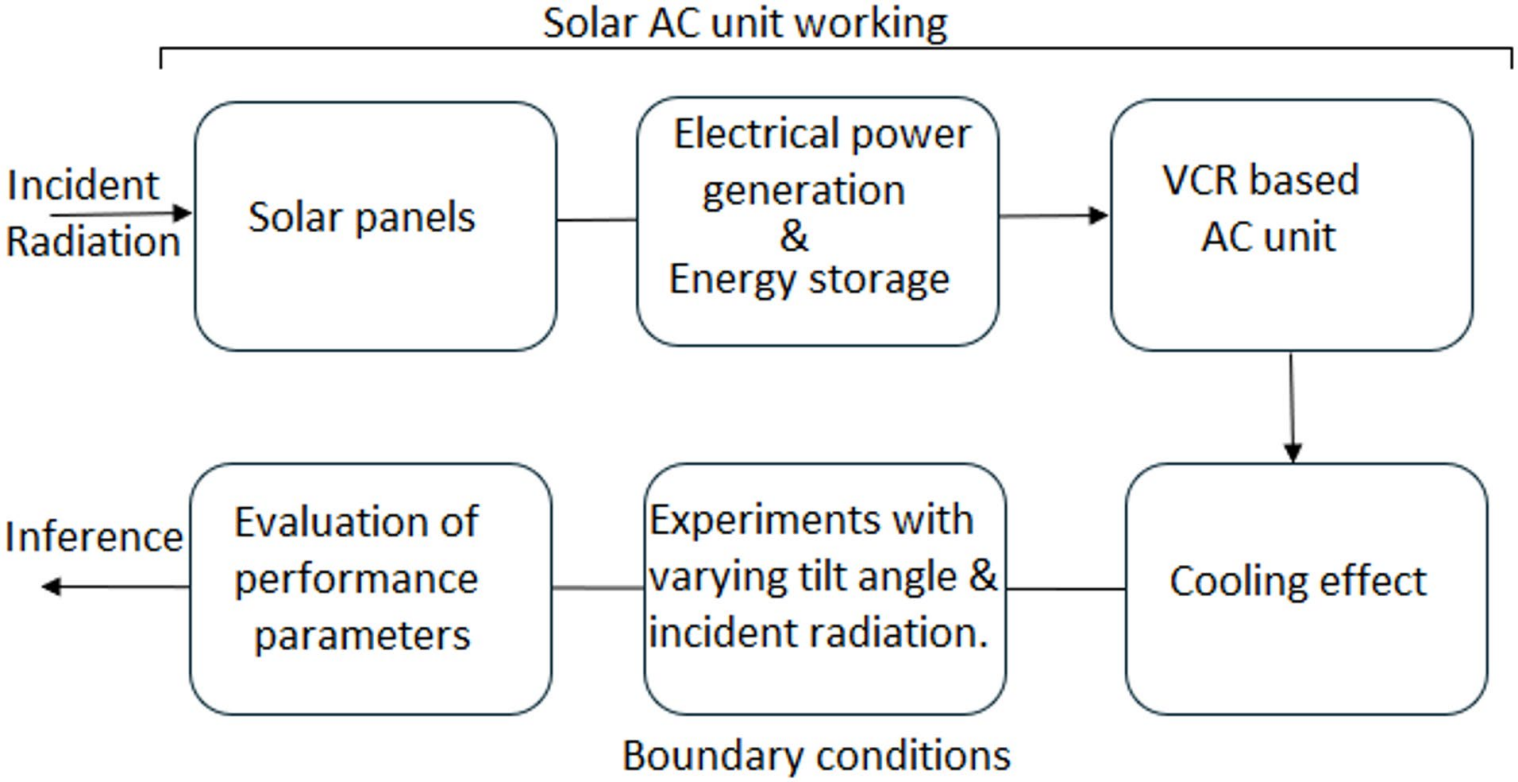
Flow chart of the solar air conditioning unit.

Figure 1 gives the methodology of the solar AC unit. In the solar AC unit, the heat energy from the sun is collected by the solar panels, and it is stored in the batteries. Power generation from photovoltaic (PV) technology is founded on the photovoltaic effect, in which semiconductor materials convert sunlight directly into electricity. In a PV cell, typically made of silicon, photons from sunlight excite electrons, freeing them from their atomic bonds. When the cell is connected to an external circuit, the flow of these electrons generates an electric current. PV cells are interconnected in series and parallel within a module to achieve the required voltage and current, and multiple modules can be assembled into arrays for greater power output. The electricity produced is direct current (DC), which can be used for DC applications, stored in batteries, or converted to alternating current (AC) via an inverter for use with standard electrical systems and grid integration. The overall efficiency of PV power generation depends on factors such as material quality, solar irradiance, temperature, and system configuration. This electric energy runs the Vapor compression refrigeration (VCR) unit compressor, which pressurizes the refrigerant vapors and operates the cycle. For the solar AC unit, performance parameters are defined by varying the tilt angles and the incident radiation.

Moisture removal rate (MRR) gives the amount of moisture removal from the air leaving the evaporator. It is given by the product of specific humidity change and air flow rate. It is given by Eq. (1). Specific humidity and air flow rate are given by the units g/kg and kg/s. Moisture Removal Rate (MRR) in a solar AC system measures the amount of moisture removed from indoor air per hour. It is vital for maintaining comfort and air quality by controlling humidity and preventing mold.

1 $$\:\dot{{m}_{c}}=\dot{{m}_{a}}({\mathrm{w}}_{1}-\:{\mathrm{w}}_{2}) [25]$$

Thermal efficiency in solar cooling systems is defined as the ratio of the useful cooling effect produced to the total solar energy incident on the collector surface. It quantifies how effectively the system converts the solar radiation absorbed over the collector area into a cooling output. Specifically, it is calculated by dividing the cooling capacity (or cooling energy delivered) by the product of the solar irradiance (incident radiation) and the collector’s surface area. Higher thermal efficiency indicates better performance, meaning more of the available solar energy is being effectively harnessed and converted into cooling. This metric is crucial for evaluating and optimizing the design and operation of solar-powered air conditioning and refrigeration systems.

The cooling effect, incident radiation, and surface have units W, W/m^2^, and m^2^.

2 $$\:{\eta\:}_{Thermal}=\frac{\dot{{Q}_{c}}}{I\:x\:{A}_{s}} [30]$$

Solar direct consumed ratio (SDCR) is defined as a ratio of the power consumed by the condenser, evaporator and compressor unit to the power supplied into the solar AC unit. All the power has the unit W. The Solar Direct Consumption Ratio (SDCR) measures the portion of solar electricity used immediately by a system without storage. A higher SDCR indicates more effective real-time use of solar energy, reducing dependence on the grid or batteries, improving efficiency, and lowering energy losses. It is especially important in solar-powered applications like air conditioning, helping optimize performance and operational costs.

3 $$\:SDCR=\frac{{\dot{W}}_{C}+{\dot{W}}_{e}+{\dot{W}}_{con}}{{P}_{battery}} [26]$$

The cooling efficiency of the AC unit is defined as the ratio of the cooling effect of the evaporator to the compressor power. Heat transfer rate and the compressor power have units W.

The cooling efficiency of an AC unit measures how effectively it converts electrical energy into cooling, commonly expressed as the Coefficient of Performance (COP) or Energy Efficiency Ratio (EER). .

4 $$\:{\eta\:}_{Cooling}=\frac{\dot{{Q}_{c}}}{\dot{W}} [25]$$

Power ratios are expressed concerning the overall battery power. It is the power consumed by individual entities to battery power. It is given by (5), (6) and (7). In a solar AC unit, compressor, evaporator, and condenser power ratios indicate component performance and overall efficiency. The compressor ratio shows its share of total power use, the evaporator ratio reflects cooling output per power input, and the condenser ratio measures heat rejection efficiency. Analyzing these ratios helps optimize energy use, detect imbalances, and maintain high cooling performance under varying solar conditions.

5 $$\:CR=\frac{{\dot{W}}_{C}}{{P}_{battery}} [27]$$

6 $$\:ER=\frac{{\dot{W}}_{e}}{{P}_{battery}} [27]$$

7 $$\:CON\:R=\frac{{\dot{W}}_{Con}}{{P}_{battery}} [27]$$

Predicted mean vote (PMV) and predicted percentage of dissatisfied (PPD) are the parameters which are defined to determine the thermal comfort inside the defined space. Equations (8) and (9) are used to represent the equations.

The Predicted Mean Vote (PMV) is an index that predicts the average thermal sensation of a group of people on a scale from − 3 (cold) to + 3 (hot), with 0 indicating thermal neutrality, based on factors such as air temperature, humidity, air velocity, clothing insulation, and metabolic rate. The Predicted Percentage of Dissatisfied (PPD) quantifies the proportion of people likely to feel thermally uncomfortable under given conditions, even when PMV is at the neutral point; at least 5% dissatisfaction is expected due to individual differences. Together, PMV and PPD are widely used to assess and optimize thermal comfort in indoor environments.

8 $$\:PMV=\left(0.303{e}^{2.1*M}+0.028\right)*[\left(M-W\right)-H-{E}_{c}-{C}_{res}-{E}_{res}] [28]$$

8a $$\:H=3.96{x10}^{-8}\:{f}_{cl}\:\left[{\left({t}_{cl}+273\right)}^{4}-{\left({t}_{r}+273\right)}^{4}\right]-{f}_{cl}\:{h}_{c}\:({t}_{cl}-{t}_{a})$$

8b $$\:{E}_{c}=3.05\:{x\:10}^{-3}\:\left[5773-6.99\:\left(M-W\right)-{P}_{a}\right]-0.42\:[\:\left(M-W\right)-58.15]$$

8c $$\:{C}_{res}=0.0014\:M\:(34-{t}_{a})$$

8d $$\:{E}_{res}=1.7\:x\:\:{10}^{-5}\:M\:(5867-{P}_{a})$$

8e $$\:{P}_{a}=RHx{P}_{v}$$

8f $$\:{h}_{c}=12.1\sqrt{V}$$

8g $$\:{t}_{r}=tx\:AF$$

9 $$\:PPD=100-95{e}^{-(0.03353*{PMV}^{4}+0.2179*{PMV}^{2})} [28]$$

where M is the metabolic rate, W/m^2^, W is the effective mechanical power, W/m^2^, H is the sensitive heat loses, W/m^2^, $\:{E}_{c}$ is the heat exchanged by the evaporation on the skin, W/m^2^, $\:{C}_{res}$ is the heat exchange by convection, W/m^2^, $\:{E}_{res}$ is the evaporation rate exchange in breathing, W/m^2^, f_cl_ is the clothing surface area factor which is equal to 1, t_cl_ is the clothing surface temperature which is equal to 29 °C t_r_ is the mean radiant temperature at 30 °C, t_a_ is the ambient temperature, RH is the relative humidity, P_v_ is the vapor pressure, P_a_ is the water vapor partial pressure, AF is the angle factor and V is the air velocity in m/s.

Solar fraction (SF) is defined as the electric energy provided by solar energy to the total electrical energy used to operate the AC unit.

10 $$\:SF=\frac{\dot{W}}{{\dot{W}}_{C}+{\dot{W}}_{e}+{\dot{W}}_{con}} [26]$$

All the equations are used to determine the performance parameters of solar AC unit with varying irradiance and solar panel angles.

### Assumptions of the study

The experimental analysis of the solar air conditioning unit was conducted under the assumption of steady-state operation, with uniform and unobstructed solar irradiance, minimal heat losses, and constant refrigerant properties. The solar panels were presumed to be clean, precisely aligned, and free from soiling, while ambient conditions and cooling load were considered stable throughout the tests. All measuring instruments were assumed to be accurately calibrated and operating within their specified accuracy limits.

## Construction, working and instrumentation

### Construction

The solar air-conditioning system consists of two main components: outdoor solar panels and an indoor unit housing a split air-conditioning system. The tilt angles of the solar panels were selected between 15° and 30°, based on established studies and practical recommendations for maximizing solar energy capture in high-irradiance regions such as Dubai. This range, aligned with the local latitude, ensures optimal energy input and cooling performance under realistic conditions without the need for mechanical tracking^33,34^.

The indoor unit integrates a vapor-compression refrigeration (VCR) cycle, including a copper tube-and-fin evaporator. A DC motor-driven blower circulates air over the evaporator coils to produce the cooling effect. The system employs a reciprocating compressor, belt-driven by a 12 V, 3500 RPM DC motor, similar to those used in Mitsubishi Fuso vehicles. The compressor compresses refrigerant via a piston mechanism, offering high reliability and pressure capability. For solar-powered operation, the system replaces the engine drive with an electric motor and integrates batteries and inverters. A dryer prevents moisture from entering the condenser, while a thermal expansion valve regulates refrigerant flow in response to temperature and pressure variations.

Solar energy captured by the PV panels is stored in a lithium-ion battery, which powers the DC motor. The motor transmits torque to the compressor via a grooved belt, enabling the piston to compress the refrigerant. A magnetic clutch controls compressor engagement, ensuring idle operation when the battery is deactivated. The system also employs a condenser set, a compact, pre-assembled unit designed for efficient heat rejection, low noise, and compatibility with various refrigerants and cooling methods. The schematic construction is shown in Fig. 1, and specifications of instruments and components are provided in Table 1.

### Working principle

**Fig. 2:**
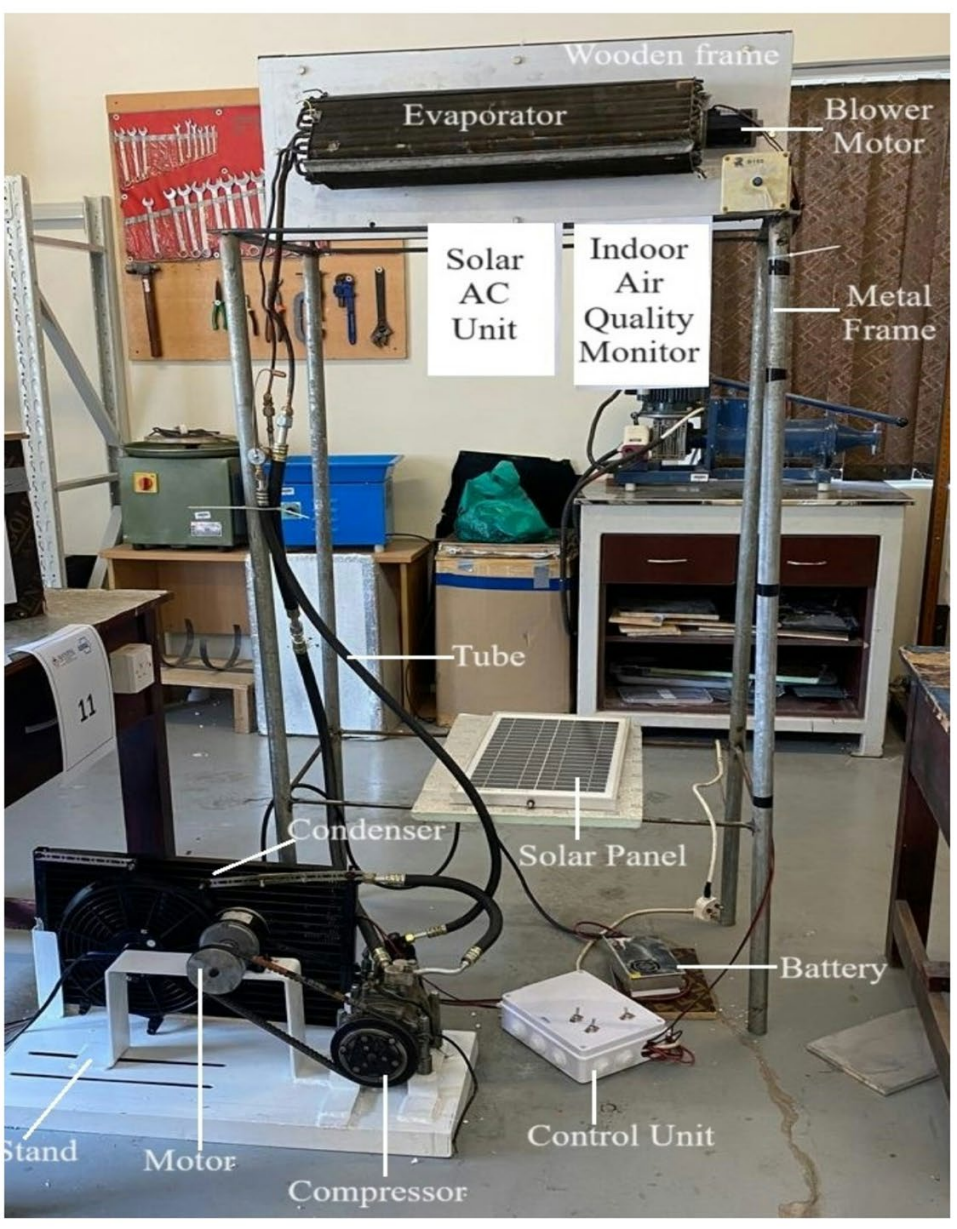
Fully constructed experimental model.

The PV panels absorb solar radiation and store energy in the batteries for consistent operation. The DC motor transmits this energy to the compressor through a belt drive, pressurizing refrigerant vapors in the reciprocating compressor. The condenser dissipates heat to the surroundings, aided by motor-driven fans, while the expansion valve precisely regulates refrigerant flow to maintain the desired temperature and pressure. The blower fan ensures uniform circulation of cooled air within the room, maintaining optimal thermal comfort. The experimental test rig is depicted in Fig. 2.

### Experimental procedure

Experiments were conducted in three separate trials to ensure accuracy and repeatability. In each trial, the solar AC operated under controlled conditions, including stable temperature, airflow, incident radiation, and tilt angle. Key performance metrics, such as humidity change, temperature variation, and power consumption, were recorded. Data from all trials were averaged, minimizing the impact of anomalies and enhancing the reliability of system performance evaluation.

### Solar radiation measurement

Solar irradiance was measured using a pyranometer, placed on a horizontal surface and carefully aligned to minimize shading and reflection errors. The instrument captured global solar radiation, including both direct and diffuse components. During peak midday hours (11:00 AM to 1:00 PM), irradiance levels occasionally exceeded 1200 W/m² due to direct sunlight and reflections from surfaces such as sand, pavements, and nearby structures. Brief peaks up to 1400 W/m² were observed, reflecting natural environmental conditions without artificial concentration. Measurements were collected at regular intervals throughout the day to accurately represent the dynamic solar input affecting the solar AC system.

### Instrumentation and material specification

**Table 1:** Component specifications.

Sl.No.	Instrument or equipment used	Details and specifications
1.	Anemometer	Measures the air velocity, Speed range = 0 to 50 m/s, Resolution = 0.05 m/s, Threshold sensitivity = 1 m/s, Accuracy = ± 0.3 m/s
2.	Hygrometer	Determines the relative humidity. Range = 0 to 99%, Resolution = 0.1%, Accuracy ± 1.0%
3.	Digital thermometer	Measures dry bulb temperature. Probe diameter = 6 mm and made of stainless steel, Accuracy of ± 0.1 °C
4.	Clampmeter	Measures instant voltage and current. Operating temperature range of 0 to 50 °C
5.	Compressor	Mitsubushi Fuso compressor piston type
6.	DC motor—compressor	12 V 3500 RPM
7.	DC motor—blower fan	12 V 2000 RPM
8.	Spal DC fan leaf and motor set	12 V
9.	Condenser set	Formula
10.	Evaporator unit	Mitsubishi Split unit Air conditioner BTU50000
11.	Expansion valve	Airkin
12.	Belt	Black belt brand V-type
13.	Pipe	Denso
14.	R134a refrigerant	Honeywell

Table 1 summarizes the specifications of the instruments and components used for constructing the solar air-conditioning system and for measuring its input and output parameters. Accurate calibration of all measuring instruments—including the thermometer, hygrometer, anemometer, and pyranometer—is essential to ensure the reliability and precision of the experimental data.

The thermometers were calibrated against a certified reference thermometer in a controlled temperature bath, validating readings across the expected measurement range. The hygrometers were verified using a standard humidity generator or a psychrometric reference under stable environmental conditions to ensure accurate relative humidity measurements. The anemometers were tested in a calibrated wind tunnel with known airflow velocities to confirm and adjust output accuracy. The pyranometer, used to measure incident solar radiation, was calibrated either against a secondary standard pyranometer traceable to the World Radiometric Reference (WRR) or through manufacturer-specified calibration constants, verified under clear-sky conditions. These calibration procedures minimize measurement errors and enhance the credibility of the solar AC performance evaluation.

In the experimental setup, thermometers and hygrometers were installed at both the inlet and outlet of the evaporator, measuring dry-bulb temperature and relative humidity of the air before and after cooling. Sensors were positioned centrally within the airflow and shielded from direct solar radiation or other external heat sources. The anemometer was placed at the fan outlet to accurately measure air velocity and ensure consistent airflow assessment. The pyranometer was installed in an unobstructed location, aligned in the same plane and tilt as the photovoltaic panels, to capture solar radiation under identical geometric and environmental conditions. This careful sensor placement guarantees that all measurements accurately reflect the true operating performance of the solar air-conditioning system.

### Uncertainty analysis

Error or uncertainty analysis quantifies the variability of the output with the change in the input. Quantification involves the estimation of statistical quantities. RSS (Root Sum Square) uncertainty analysis provides a more accurate estimate of overall uncertainty by combining independent error sources using the square root of their squared sums. This method avoids overestimating errors, assuming random and uncorrelated uncertainties, and is essential in engineering and science for evaluating result reliability, quality control, and informed decision-making.

Let Y_G_ is the error, G is the function, X & Y are independent, and intervals. Equation (11) is root sum of squares method used to calculate the uncertainty while measuring and calculating the performance parameter. Error analysis is evaluated and presented in the Table 2.

11 $$\:{{\mathrm{Y}}_{G}\:=\:\left[{\left(\frac{\delta\:G}{\delta\:{X}_{1}}\:{Y}_{1}\right)}^{2}+\:{\left(\frac{\delta\:G}{\delta\:{X}_{2}}\:{Y}_{2}\right)}^{2}+{\left(\frac{\delta\:G}{\delta\:{X}_{3}}\:{Y}_{3}\right)}^{2}+\dots\:\dots\:\dots\:\dots\:.\:+{\left(\frac{\delta\:G}{\delta\:{X}_{4}}\:{Y}_{n}\right)}^{2}\right]}^{0.5} [25] [28]$$

Condensation rate is given by $\:\text{}\text{}\dot{{m}_{c}}\mathrm{=}\dot{{m}_{a}}\left({\mathrm{w}}_{\mathrm{2}}\mathrm{-}{\mathrm{w}}_{\mathrm{1}}\right)$= $\:\dot{{\mathrm{m}}_{\mathrm{a}}}\Delta\mathrm{W}$.

12 $$\text{Uncertainty in condensation rate},\:\frac{\partial\:\left(\dot{{m}_{W}}\right)}{\dot{{m}_{W}}}=\sqrt{{\left(\frac{\partial\:\dot{{m}_{a}}}{\dot{{m}_{a}}}\right)}^{2}\:+{\left(\frac{\partial\:\Delta\mathrm{W}}\Delta{\mathrm{W}}\right)}^{2}} [25]$$

Thermal efficiency is given by $\:{\eta\:}_{Thermal}=\frac{\dot{{Q}_{c}}}{I\:x\:{A}_{s}}$.

13 $$\text{Uncertainty in thermal efficiency, }\:\frac{\partial\:\left({\eta\:}_{Thermal}\right)}{{\eta\:}_{Thermal}}=\sqrt{{\left(\frac{\partial\:\dot{{Q}_{c}}}{\dot{{Q}_{c}}}\right)}^{2}\:+{\left(-\frac{\partial\:I}{I}\right)}^{2}+{\left(-\frac{\partial\:{A}_{s}}{{A}_{s}}\right)}^{2}} [25]$$

Solar direct consumed ratio is given by $\:SDCR=\frac{{\dot{W}}_{C}+{\dot{W}}_{e}+{\dot{W}}_{con}}{{P}_{battery}}$.

14 $$\text{Uncertainty of SDCR, }\:\frac{\partial\:\left(SDCR\right)}{SDCR}=\sqrt{{\left(\frac{\partial\:{\dot{W}}_{C}}{{\dot{W}}_{C}}\right)}^{2}{+\left(\frac{\partial\:{\dot{W}}_{e}}{{\dot{W}}_{e}}\right)}^{2}+{\left(\frac{\partial\:{\dot{W}}_{cond}}{{\dot{W}}_{cond}}\right)}^{2}+{\left(-\frac{\partial\:{P}_{battery}}{{P}_{battery}}\right)}^{2}} [25]$$

Cooling efficiency is given by $\:{\eta\:}_{Cooling}=\frac{\dot{{Q}_{c}}}{\dot{W}}$.

15 $$\text{Uncertainty of cooling efficiency, }\:\frac{\partial\:\left({\eta\:}_{Cooling}\right)}{{\eta\:}_{Cooling}}=\sqrt{{\left(\frac{\partial\:\dot{{Q}_{c}}}{\dot{{Q}_{c}}}\right)}^{2}{+\left(-\frac{\partial\:\dot{W}}{\dot{W}}\right)}^{2}} [25]$$

Solar fraction is given by $\:SF=\frac{\dot{W}}{{\dot{W}}_{C}+{\dot{W}}_{e}+{\dot{W}}_{con}}$.

16 $$\text{Uncertainty of SF, }\:\frac{\partial\:\left(SF\right)}{SF}=\sqrt{{\left(\frac{\partial\:\dot{W}}{\dot{W}}\right)}^{2}{+\left(-\frac{\partial\:{\dot{W}}_{C}}{{\dot{W}}_{C}}\right)}^{2}+{\left(-\frac{\partial\:{\dot{W}}_{e}}{{\dot{W}}_{e}}\right)}^{2}+{\left(-\frac{\partial\:{\dot{W}}_{con}}{{\dot{W}}_{con}}\right)}^{2}} [25]$$

**Table 2:** Error values of solar AC unit.

Sl.no	Variable	Percentage uncertainty or error
1.	Condensation rate of moisture removal rate	1.87
2.	Thermal efficiency	1.56
3.	Solar direct consumed ratio	2.31
4.	Cooling efficiency	2.25
5.	Solar fraction	1.45

## Results and discussions

Experiments are performed on the solar-powered AC unit with the variation of the incident radiation and the angle of the solar panels at a fixed air velocity.Table 3 shows the experimental parameters of the present study. The results and discussion section is mainly divided into 2 sections.

(i) Variation of parameters with the incident radiation and solar angle.
(ii) Energy consumption and thermal comfort conditions.

**Table 3:** Experimental parameters.

Sl.No.	Type of refrigerant	Parameters variation	Input and output parameters	Performance parameters	Aim of study
1.	R22	Air velocity: 6.5 m/s Solar angle: 15º, 20º, 25º, and 30º Incident radiation: 700 to 1400 W/m^2^ Mass flow rate of the refrigerant (kg/s) = 0.012 kg/s Timeline of experiments: 9:00, 11:00, 13:00, 15:00, and 17:00 h	Dry bulb temperature (DBT), air velocity, relative humidity, incident radiation, Solar panel angle, energy consumption	Moisture removal rate, thermal efficiency, solar direct consumed ratio, cooling efficiency, solar fractions, power ratios, Predicted mean vote and predicted percentage of dissatisfaction	To evaluate the optimum incident radiation and the solar panel angle to obtain maximum performance of the solar ac unit and maintain thermal comfort

(i) Variation of parameters with the incident radiation and solar angle.

### Moisture removal rate (g/s)

**Fig. 3:**
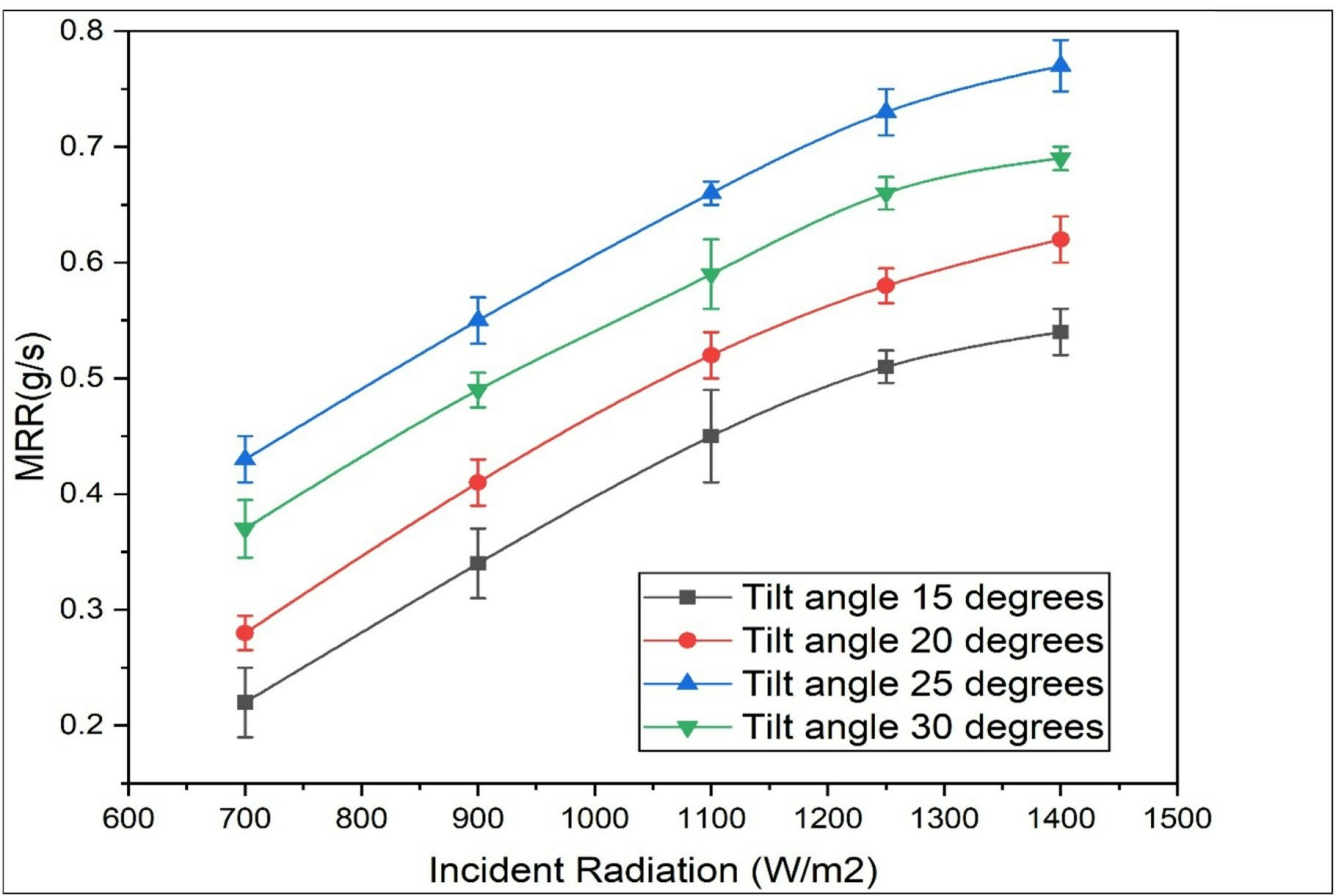
Variation of MRR with incident solar radiation and tilt angles.

The moisture removal rate (MRR) refers to the amount of moisture extracted from the air over a specific period. It has been observed that as the intensity of solar radiation increases, the temperature of the air drops more significantly when the blower velocity remains constant. This results in an increased cooling effect within the evaporator, consequently increasing the enthalpy difference of the air. Subsequently, the ΔW (specific humidity difference) increases, leading to an upsurge in the MRR as shown in Fig. 3. The increase in mass removal rate (MRR) with incident radiation occurs because higher solar radiation provides more thermal energy, which enhances evaporation or melting processes, thereby increasing the rate at which material is removed. The performance of the solar air conditioning system is also notably affected by the tilt angle of the solar collector. It is crucial to determine the optimal tilt angle that allows for the maximum capture of solar radiation, thereby ensuring effective performance.

Regarding the solar tilt angle, MRR initially rises from 15° to 20° because this range optimizes the angle at which solar rays strike the surface, maximizing the effective absorption of solar energy. Beyond 20°, the tilt angle reduces the direct exposure to sunlight due to geometric factors and possible shading effects, leading to less absorbed energy and thus a decrease in MRR. In essence, the MRR depends on both the intensity of available solar energy and the orientation of the surface to capture that energy most effectively. When the radiation is increased from 700 W/m^2^ to 1400 W/m^2^ the MRR is found to be increased by 81.39% for a tilt angle of 25 degrees and similarly, when the tilt angle is increased from 15 degrees to 25 degrees MRR is surged by 56%.

### Thermal efficiency

**Fig. 4:**
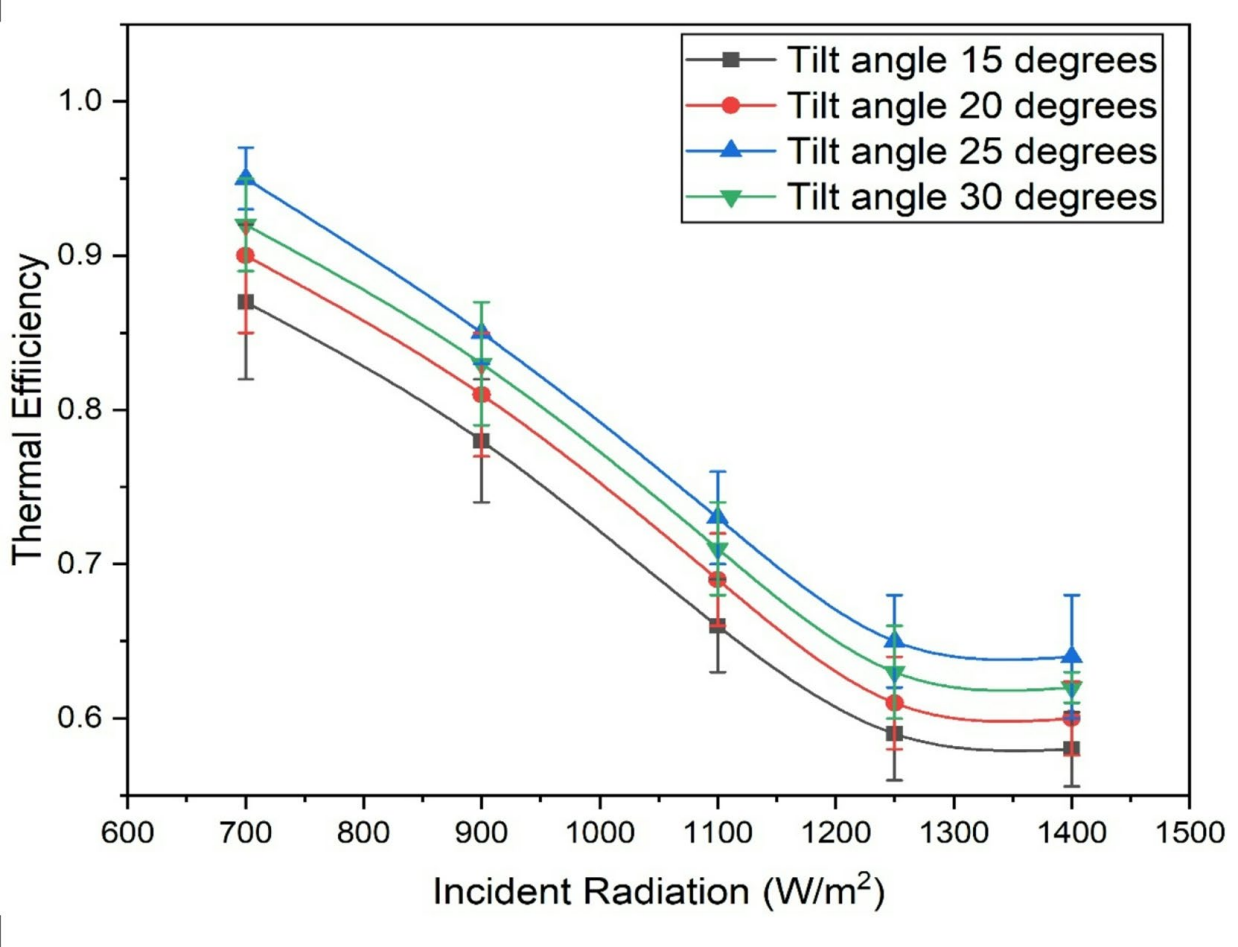
Variation of thermal efficiency with incident solar radiation and tilt angles.

The thermal efficiency is a measure of the cooling effect produced to the incident energy expressed in kilowatts (kW). It is observed that as the solar radiation increases, the thermal efficiency decreases as shown in Fig. 4. This is due to the higher losses incurred with higher solar radiation. Although the cooling effect also increases with the rise in solar radiation, the rate of increase is lower than the total energy input, leading to a reduction in thermal efficiency. Also, at higher radiation levels, more energy is absorbed than can be effectively utilized by the system, leading to greater thermal losses through convection, radiation, and heat dissipation. This reduces the proportion of useful cooling output relative to the input energy, lowering efficiency. These results were compared with literature values from Villarini et al.^1^, Li et al.^30^ and Sulaiman et al.^35^ and the present unit yielded higher performance.

Additionally, research on the solar tilt angle indicates that increasing the tilt angle from 15 degrees to a higher value also increases the thermal efficiency for a specific level of solar radiation. When the tilt angle is too low, the rays are unable to strike the collector perpendicularly, resulting in reduced energy capture and decreased energy input to the solar AC system, thus lowering the thermal efficiency. Similarly, when the tilt angle is too high, it has similar effects on the energy input and thermal efficiency. The current findings suggest that a tilt angle of 25 degrees yields the highest thermal efficiency. When the radiation is increased from 700 W/m^2^ to 1400 W/m^2^ the thermal is found be decreased by 32.29% for a tilt angle of 25 degrees and similarly when the tilt angle is increased from 15 degrees to 25 degrees thermal efficiency is increased by 14.03%.

### Solar COP

The coefficient of performance (COP) is a measure of the cooling effect observed for the air in relation to the energy input. When solar radiation is at a lower level, the input energy is reduced, which minimizes losses and results in a moderate cooling effect because of a lower denominator value, leading to higher COP values. However, as solar radiation increases, the corresponding losses also increase. Despite the increasing cooling effect, the rate of increase in thermal energy input surpasses the increase in the obtained cooling effect, ultimately reducing the COP of the system. Higher temperatures increase the kinetic energy of refrigerant molecules, leading to higher rates of evaporation and condensation within the vapor compression cycle. However, excessive heat can cause inefficiencies such as increased refrigerant pressure and temperature beyond optimal operating ranges, resulting in greater internal friction, heat losses, and less effective cooling per unit of input energy. These molecular-level inefficiencies reduce the overall cooling output relative to the energy consumed, causing the solar COP to decrease as incident radiation increases. The solar COP obtained by the present study are at par with the literatures Villarini et al.^1^, Li et al.^30^.

**Fig. 5:**
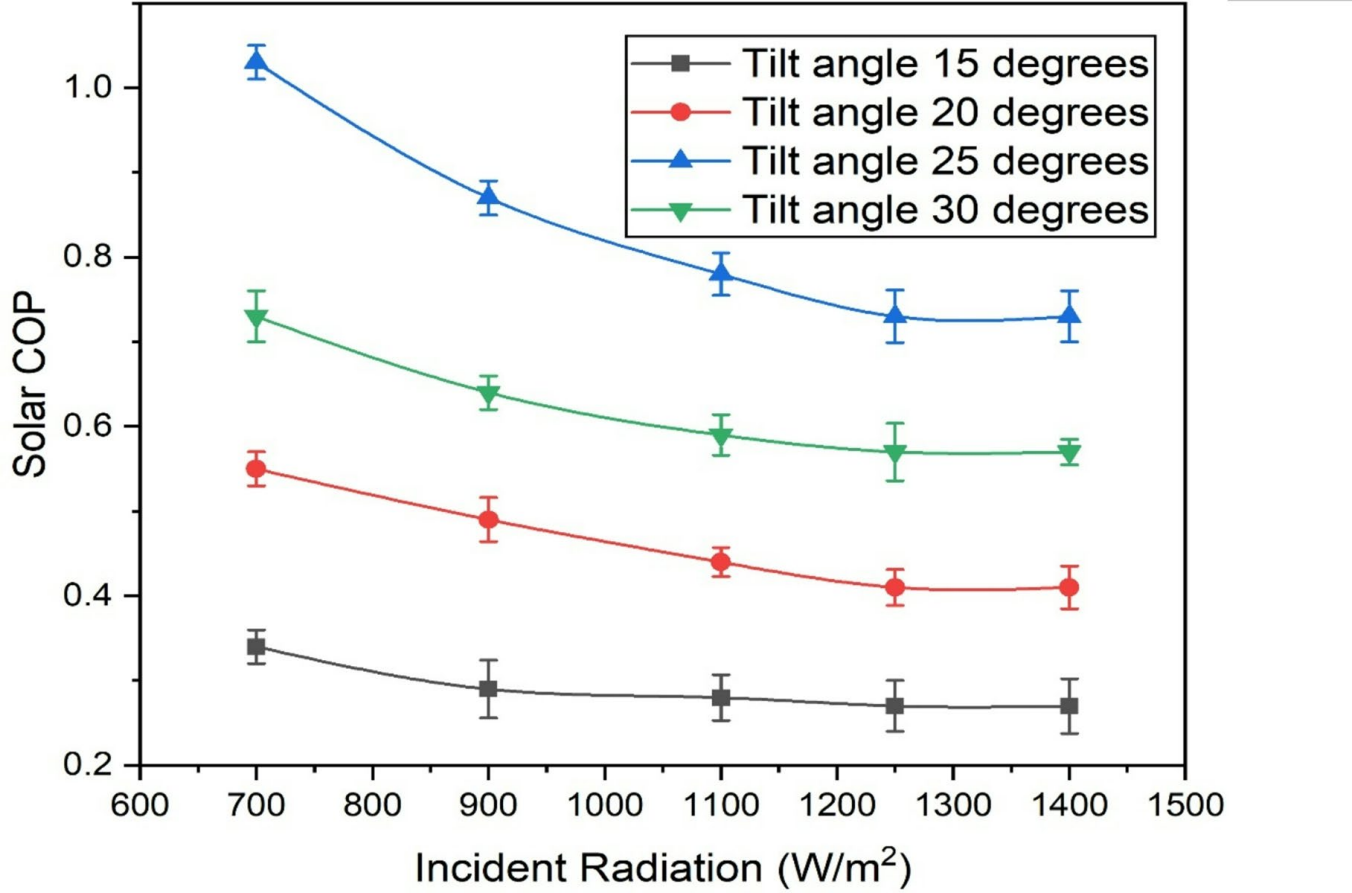
Variation of solar COP with incident solar radiation and tilt angles.

Lower tilt angles result in a lower COP, as the observed cooling effect itself will be lower. As the tilt angle increases up to 25 degrees, the COP increases. However, further higher tilt angles result in a decline in COP due to the reduced thermal energy collected by the collector which is clearly visible in Fig. 5. Maximum COP was noticed when the solar radiation was for a tilt angle of 25 degrees. It reduced by 70.58% and 25.71% when the tilt angle is reduced to 15 degrees and solar radiation was increased to 1400 W/m^2^.

Considering the room Size: 10 ft × 10 ft × 10 ft (volume: 1000 ft³ or 28.3 m³). with 10 Occupants, 2 windows (assume standard size: 3 ft × 4 ft each, the split of the total cooling load required is.

Occupants: 1000 W.

Solar Heat Gain: 200 W (after shading/insulation).

Lighting: 93 W.

Equipment: 100 W.

Adjusted Total Heat Gain: Total Heat Gain = 1000 + 200 + 93 + 100 = 1293 W “(≈ 1.3” kW” Based on these values, the maximum cooling load for the system is determined to be 1.3 kW. The above volume was significantly cooled, and the required temperature is maintained.

### Solar direct consumed ratio (SDCR)

**Fig. 6:**
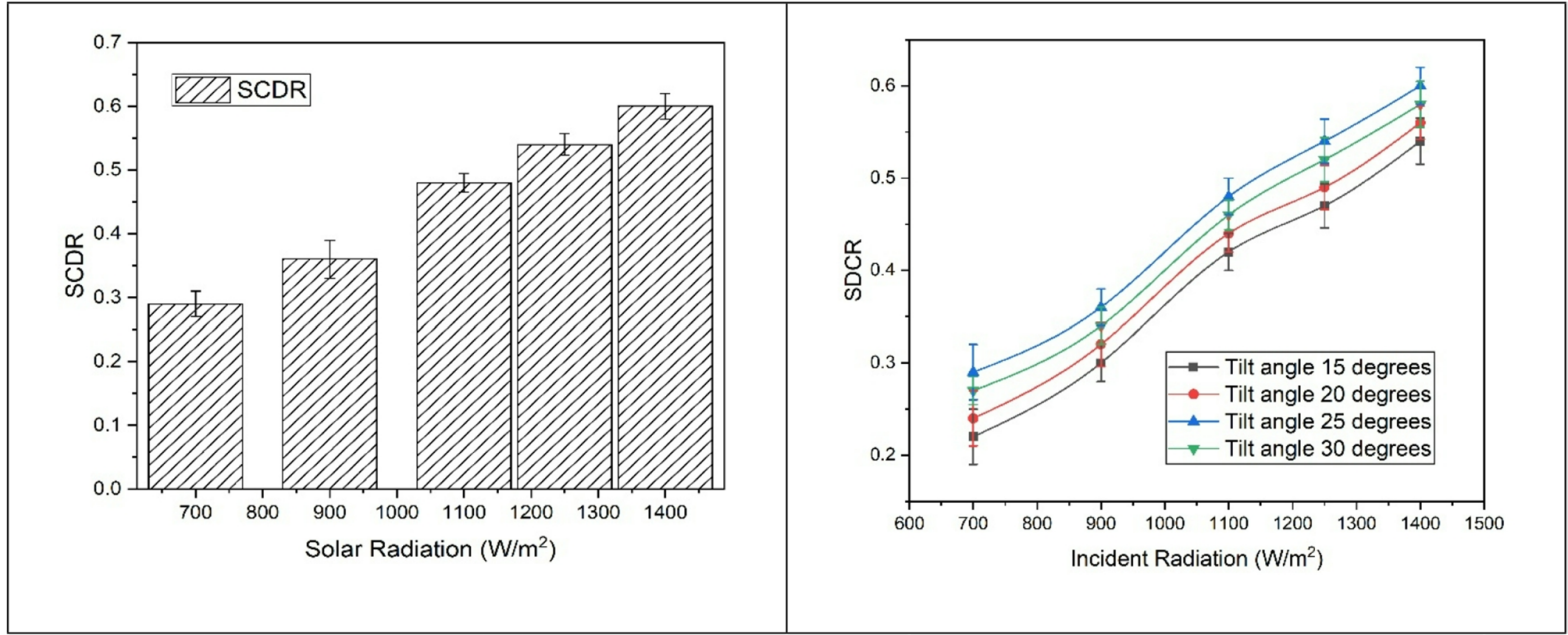
Variation of SCDR with incident solar radiation and tilt angles.

As the incident radiation increases, the solar-direct consumed ratio (SDCR) also increases, as shown in Fig. 6. For a specific tilt angle, an increase in incident radiation results in more energy being received from the collector. This, in turn, increases the energy utilized by the combination of the motor and blower for running the compressor and fan. The combination efficiently works by drawing more energy from the collector, leading to higher SDCR values.

As incident solar radiation intensifies, more photons are absorbed by the solar collector, increasing the thermal energy available to the working fluid. At the molecular level, this energy raises the kinetic energy of fluid molecules, enhancing evaporation rates and improving heat transfer efficiency within the refrigeration cycle. The increased molecular activity allows the system to convert a larger fraction of the absorbed solar energy directly into useful cooling work. Consequently, a higher proportion of the incident solar energy is effectively consumed in driving the air conditioning process, leading to an increase in the solar direct consumed ratio. This trend can be observed from the literature Villarini et al.^1^, Li et al.^30^, Sulaiman et al.^35^, and Alahmer et al.^36^. The variation of SCDR with the tilt angle is shown in Fig. 6. It is observed that for the minimum tilt angle, SCDR remains at its lowest, and as the tilt angle is increased, it reaches a maximum corresponding to a tilt angle of 25 degrees. As the tilt angle increases, the energy received by the collector increases, consequently leading to an increase in the energy received by the solar AC system and, in turn, increasing the SCDR. From the current study it is revealed that when the solar radiation was increased from 700 to 1400 W/m^2^ SCDR increased by 103.44% for the best tilt angle. Further for the minimum radiation of 700 W/m2, when the tilt angle is increased from 15 to 25 degrees it increased by 15.38% and for 1400 W/m^2^ the values being 106.89%.

(ii) Energy consumption and thermal comfort conditions.

### Power ratios

**Fig. 7:**
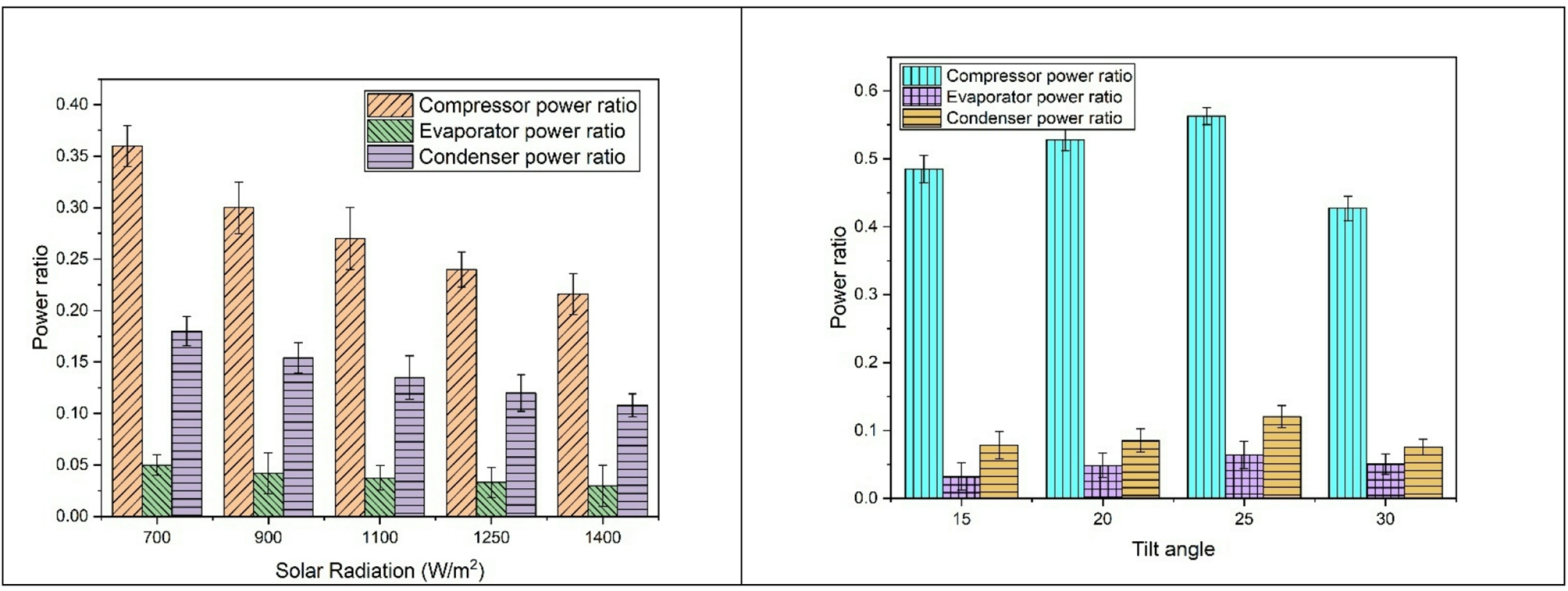
Variation of power ratios with incident solar radiation and tilt angles.

The graph in Fig. 7. demonstrates how the power ratios vary with different levels of solar radiation and tilt angles. It shows that the compressor requires the highest power, followed by the condenser and the evaporator. As the incident radiation increases, the collector and other solar AC components, such as the compressor and evaporator, receive more energy, leading to higher power fractions for each component. Specifically, when the solar radiation increases from 700 W/m^2^ to 1400 W/m^2^, the compressor, evaporator, and condenser power ratios decreases by 31.42%, 22.22%, and 20% respectively.

The figure also shows how power ratios vary with the tilt angle. It is observed that power ratios increase as the tilt angle is increased from a lower value of 15 degrees. For this orientation of the collector, excess losses will decrease the energy received by the collector and energy received by the solar AC components. As the tilt angle is slowly increased from this value, losses will be minimized and energy taken by the compressor, evaporator, and the condenser will be increased. Hence, their fractions also seem to be on an increasing trend. However, increasing the tilt angle above 25 degrees results in negated benefits, and once again, performance shows a negative trend, reducing the power ratios.

### Variation of thermal comfort parameters. (PMV and PPD values)

**Fig. 8:**
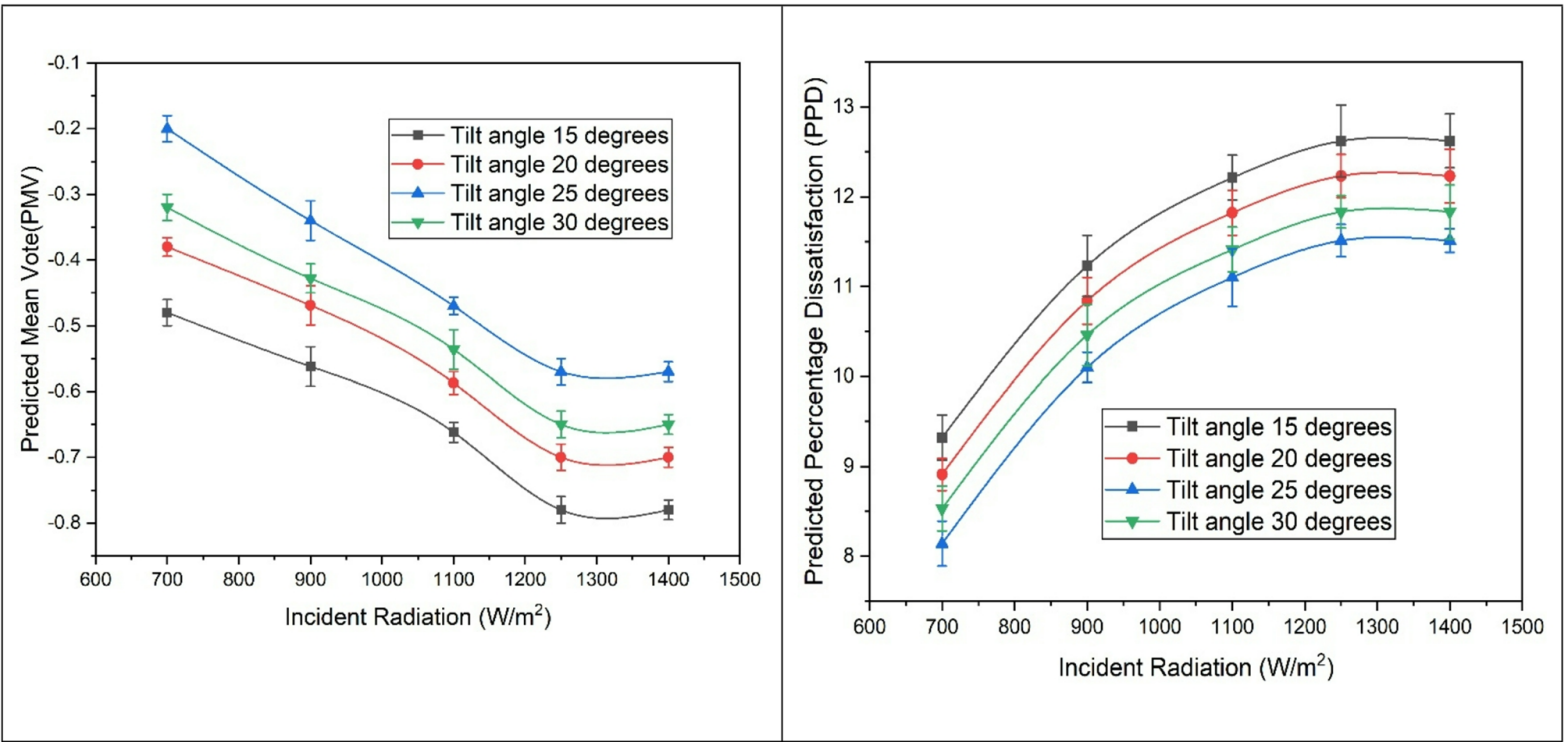
Variation of PMV and PPD values with solar radiation and tilt angles.

The graph in Fig. 8 shows the variation of PMV with solar radiation. For optimal thermal comfort, PMV values should fall within the range of − 3 to + 3, with values close to zero indicating the best conditions. According to the data from the current study, all values are within the specified range and are close to zero, indicating thermal neutrality. As solar radiation increases, the PMV values shift slightly towards the negative side, indicating a it is slightly deviating away from the thermal comfort conditions. This is because due to high incident radiation, temperature and humidity inside the room is higher which is away from the standard thermal comfort range. Although. the values move slightly towards the negative side, they still fall within the specified limits for thermal comfort conditions. Similar values are also observed with an increase in tilt angle, with 25 degrees showing the best results.

The Predicted Percentage of Dissatisfied (PPD) values are used to assess thermal comfort in indoor environments. These values indicate the percentage of people dissatisfied by the thermal conditions. As the input solar radiation intensity increases, the PPD values also increase, reaching a maximum value of 12, which is very low according to the literature. This indicates a lower percentage of dissatisfaction for the range of solar radiation considered in the present work. The variation of PPD with a tilt angle of 15 degrees is found to be the highest and decreases as the tilt angle is increased. At 25 degrees, it reaches a minimum, showing the optimum tilt angle. Upon further increasing the angle, PPD values are also found to be higher. Maximum results obtained in the solar air conditioning unit if given by Table 4.

**Table 4:** Maximum results obtained in the solar air conditioning unit.

Sl.No	Performance parameter	Maximum value obtained
1.	Moisture removal rate (MRR)	0.77 g/s
2.	Thermal efficiency	96%
3.	Solar direct consumed ratio (SDCR)	0.6
4.	Solar COP	1.1
5.	Compressor power ratio	0.37
6.	Evaporator power ratio	0.04
7.	Condenser power ratio	0.18
8.	Predicted mean vote (PMV)	-0.2
9.	Predicted percentage dissatisfaction (PPD)	12.5

### Validation of the results

The results obtained from the current unit are validated against those reported in the literature by considering three key performance parameters: solar direct consumed ratio (SDCR), coefficient of performance (COP), and thermal efficiency. In this study, the unit achieved maximum values of 0.6 for SDCR, 1.1 for COP, and 96% for thermal efficiency. When compared with values from Villarini et al.^1^, Li et al.^30^, Sulaiman et al.^35^, and Alahmer et al.^36^, the thermal efficiency of the present unit is higher than all reported figures. Although Li et al.^30^ reported a slightly higher SDCR of 0.7 and Sulaiman et al.^35^ recorded a higher COP of 2.8, the overall performance of the current system is either superior to or on par with most literature values.This improvement can be attributed to the optimized combination of tilt angle and incident radiation, which ensures maximum solar energy utilization, as well as the effective integration of the cooling cycle components. Table 5. gives the details supporting the validation.

: 

**Table 5:** Comparison of the present results with that of existing solar AC units.

Sl. No	Authors name	Unit	Input parameters	SDCR	COP	Thermal efficiency (%)
1.	Present study	Solar PV air conditioner with the variation of the tilt angle and the incident radiation	Air velocity: 6.5 Incident radiation vatiation:700–1400 W/m^2^ Solar panel angle: 15 to 30º	0.6	1.1	96
2.	Villarini et al.^1^	Solar Organic Rankine Cycle trigenerative systems	Time line of experiment: 6 AM to 6 PM	0.5	0.7	77
3.	Li et al.^26^	Photovoltaic thermoelectric air-conditioning system	Irradiance:100 to 1000 W/m^2^	0.7	0.37	75
4.	Sulaiman et al.^29^	Solar powered off-grid air conditioning system with natural refrigerant	Condenser temp variation: 36 to 55 °C Refrigerants used: R134a, R22, R410A, R32, R290, R600a.	-	2.8	56
5.	Alahmer et al.^30^	Dynamic and economic investigation of a solar thermal-driven two-bed adsorption chiller	Timeline of experiment: 6 a.m. to 6 p.m.	0.65	0.7	-

## Conclusions

The present study examined the performance of a solar air conditioning unit under varying levels of incident solar radiation and panel tilt angles, yielding the following key conclusions.

1. An increase in solar radiation intensity enhances the evaporator’s cooling effect by lowering the air temperature and increasing the air’s enthalpy difference, resulting in a peak moisture removal rate of 0.78 g/s. However, higher solar radiation also leads to greater thermal losses, causing a reduction in overall thermal efficiency.
2. Increasing the tilt angle beyond 15º enhances thermal efficiency, as lower angles reduce energy capture due to oblique solar incidence. A tilt angle of 25º yields the highest thermal efficiency.
3. The system achieved a maximum COP of 1.1 and SCDR of 0.6. Lower tilt angles reduced performance due to weaker cooling, while increasing the tilt up to 25° improved both COP and SCDR. Beyond 25°, decreased thermal input led to a drop in COP.
4. Increasing the tilt angle up to 25° minimizes losses and boosts energy absorption by the compressor, evaporator, and condenser, with maximum power ratios of 0.36, 0.04, and 0.17, respectively. Beyond 25°, power ratios decline due to reduced efficiency.
5. With an incident radiation of 1400 W/m², the PMV of − 2.1 and PPD of 12.7 indicate the solar AC unit effectively maintains thermal comfort in the designated space.
6. The system’s use of renewable energy reduces reliance on conventional sources, lowers environmental impact, and supports global sustainability goals, especially SDGs.

Solar air conditioning units present a range of advantages, including cost-effectiveness, sustainability, and high energy efficiency. By harnessing solar energy, they reduce dependence on conventional power sources, lower environmental impact, and ensure consistent thermal comfort within indoor spaces. This initiative aligns with and advances Sustainable Development Goals (SDGs) 7 and 11 by promoting access to clean, renewable energy and supporting the development of sustainable, resilient living environments for both residential and industrial applications.

### Scope for future work

Here are some potential scope for future work on solar air conditioning systems, presented below.

1. Integration of thermal comfort-based control strategies: Implement PMV/PPD-driven smart controllers that adjust fan speed, compressor operation, or airflow to maintain optimal comfort while minimizing energy consumption.
2. Hybrid cooling enhancements: Investigate the integration of phase-change materials (PCM) or liquid cooling with the solar AC system to further enhance PV efficiency and overall cooling performance.
3. Scaling up for larger spaces or multi-room applications: Study the performance of multi-unit solar AC systems or modular setups for residential and commercial applications under extreme irradiance conditions.

### Cost analysis and payback period

In Dubai’s hot desert climate, where cooling accounts for a significant portion of electricity consumption, solar air conditioning (solar AC) offers clear long-term advantages over conventional grid-powered AC despite higher initial costs for photovoltaic panels, inverters, and mounting systems. Considering the abundant annual sunshine and high solar irradiance, solar AC can meet most or all of its daytime energy demands, unlike regular AC, which depends entirely on costly grid electricity. Maintenance requirements are comparable, with solar AC requiring only periodic panel cleaning. The costs of a 1-ton capacity conventional AC and solar AC, including all mountings, are approximately 1000 AED and 1300 AED, respectively. Similarly, the electricity consumption costs per hour are 1.84 AED for conventional AC and 1.9 AED for solar AC. Based on these differences, the payback period for the solar AC unit is estimated to be just 4 h. Table 6 provides detailed information on the costs and electricity consumption of both solar and conventional AC units.

**Table 6:** Cost and electricity consumption by solar AC compared to conventional AC.

	Solar AC unit	Conventional AC unit
Initial cost	1300 AED	1000 AED
Electricity consumption cost per hour	1.19 AED/h	1.84 AED/h
Payback period	4 months	

## Data Availability

The data that support the findings of this study are openly available in Mendeley Data at DOI: 10.17632/ggnpp9wt48.1.

## Abbreviations

SRRR: Solar radiation reception rate
PTSP: Poly tilted segmented panel
ISRPI: Incident solar radiation presiction index
DASH: Dual angle solar harvest
PMV: Predicted mean vote
PPD: Predicted percentage of dissatisfied
CON R: Condenser power ratio
COP: Coefficient of performance
CR: Compressor power ratio
DBT: Dry bulb temperature (ᵒC)
DC: Direct current
ER: Evaporator power ratio
SPV: Solar photovoltaic
PV: Photovoltaic
RH: Relative humidity
SDCR: Solar direct consumed ratio
SF: Solar fraction
VCR: Vapor compression refrigeration
WBT: Wet bulb temperature (°C)

## Variables

$\dot{W}$: Power supplied to the compressor (W)
$\dot{{m}_{a}}$: Mass flow rate of air (kg/s)
$$\dot{{m}_{c}}$$: Condensation rate (g/s)
${W}_{1}$: Specific humidity at the inlet (g/kg)
${W}_{2}$: Specific humidity at the outlet (g/kg)
${\dot{Q}}_{C}$: Cooling effect (Watts)
P_battery_: Power consumed by battery (Watts)
$${\eta }_{Thermal}$$: Thermal efficiency (%)
$${\eta }_{Cooling}$$: Cooling efficiency (%)
I: Incident radiation (W/m^2^)
A_s_: Surface area of the collector (m^2^)
$${COP}_{Solar}$$: Solar COP
${\dot{W}}_{C}$: Power consumed by compressor (Watts)
${\dot{W}}_{e}$: Power consumed by evaporator (Watts)
${\dot{W}}_{con}$: Power consumed by condenser (Watts)
M: Metabolic rate (W/m^2^)
H: Sensitive heat loss (W/m^2^)
W: Mechanical power (W/m^2^)
E_c_: Heat exchange due to evaporation of skin (W/m^2^)
C_res_: Heat exchange by convection (W/m^2^)
E_res_: Heat exchange by evaporation (W/m^2^)
f_cl_: Clothing surface area factor which is equal to 1
t_cl_: Clothing surface temperature, ⁰C
t_r_: Mean radiant temperature, ⁰C
t_a_: Ambient temperature, ⁰C
RH: Relative humidity , %
P_v_: Vapor pressure, Pa
P_a_: Water vapor partial pressure, Pa
AF: Angle factor
V: Air velocity in m/s.
X: Independent variable
Y: Uncertainty intervals
G: Uncertainty function
Y_G_: Total uncertanity
$\frac{\partial (\dot{{m}_{W}})}{\dot{{m}_{W}}}$: Uncertainty of condensation rate
$\frac{\partial ({\eta }_{Thermal})}{{\eta }_{Thermal}}$: Uncertainty of thermal efficiency
$\frac{\partial (SDCR)}{SDCR}$: Uncertainty of solar direct consumed ratio
$\frac{\partial ({\eta }_{Cooling})}{{\eta }_{Cooling}}$: Uncertainty of cooling efficiency
$\frac{\partial (SF)}{SF}$: Uncertainty of solar fraction

## Subscripts

1: Inlet
2: Outlet
a: Air
c: Cooling
C: Compressor
Con: Condenser
e: Expansion
G: Uncertainty
v: Vapor
cl: Cloth
res: Exchange
s: Surface
r: Radiant
